# Aerobic Threshold Identification in a Cardiac Disease Population Based on Correlation Properties of Heart Rate Variability

**DOI:** 10.3390/jcm10184075

**Published:** 2021-09-09

**Authors:** Bruce Rogers, Laurent Mourot, Thomas Gronwald

**Affiliations:** 1 College of Medicine, University of Central Florida, 6850 Lake Nona Boulevard, Orlando, FL 32827-7408, USA; 2 EA3920 Prognostic Factors and Regulatory Factors of Cardiac and Vascular Pathologies, Exercise Performance Health Innovation (EPHI) Platform, University of Bourgogne Franche-Comté, 21000 Besançon, France; laurent.mourot@univ-fcomte.fr; 3 Division for Physical Education, National Research Tomsk Polytechnic University, 634050 Tomsk, Russia; 4 Department of Performance, Neuroscience, Therapy and Health, MSH Medical School Hamburg, Faculty of Health Sciences, University of Applied Sciences and Medical University, 20457 Hamburg, Germany; Thomas.Gronwald@medicalschool-hamburg.de

**Keywords:** ventilatory threshold, HRV, detrended fluctuation analysis, DFA a1, endurance exercise

## Abstract

An index of heart rate (HR) variability correlation properties, the short-term scaling exponent alpha1 of detrended fluctuation analysis (DFA a1) has shown potential to delineate the first ventilatory threshold (VT1). This study aims to extend this concept to a group of participants with cardiac disease. Sixteen volunteers with stable coronary disease or heart failure performed an incremental cycling ramp to exhaustion PRE and POST a 3-week training intervention. Oxygen uptake (VO_2_) and HR at VT1 were obtained from a metabolic cart. An ECG was processed for DFA a1 and HR. The HR variability threshold (HRVT) was defined as the VO_2_, HR or power where DFA a1 reached a value of 0.75. Mean VT1 was reached at 16.82 ± 5.72 mL/kg/min, HR of 91.3 ± 11.9 bpm and power of 67.8 ± 17.9 watts compared to HRVT at 18.02 ± 7.74 mL/kg/min, HR of 94.7 ± 14.2 bpm and power of 73.2 ± 25.0 watts. Linear relationships were seen between modalities, with Pearson’s r of 0.95 (VO_2_), 0.86 (HR) and 0.87 (power). Bland–Altman assessment showed mean differences of 1.20 mL/kg/min, 3.4 bpm and 5.4 watts. Mean peak VO_2_ and VT1 did not change after training intervention. However, the correlation between PRE to POST change in VO_2_ at VT1 with the change in VO_2_ at HRVT was significant (r = 0.84, *p* < 0.001). Reaching a DFA a1 of 0.75 was associated with the VT1 in a population with cardiac disease. VT1 change after training intervention followed that of the HRVT, confirming the relationship between these parameters.

## 1. Introduction 

Training zone classification is a cornerstone for exercise intensity distribution study and implementation [1]. Although there may be different schools of thought on what type of distribution is “optimal” (polarized vs. pyramidal vs. threshold) all models are defined by having the major portion of training done at a low exercise intensity in the various fields of application [1]. Examination of training intensity distribution in other subject populations such as those with ischemic heart disease or congestive heart failure also indicates the importance of proper workload modulation and low-intensity (aerobic) exercise [2,3,4]. To identify these training zones, a set of boundaries have been developed. In the classic three-zone model, separation is determined by lactate or gas exchange markers, with the lowest-intensity zone (zone 1) below the aerobic threshold as denoted by the first lactate (LT1) or ventilatory threshold (VT1) [5,6]. Unfortunately, both gas exchange [7] and lactate threshold [8,9] methods of identification are subject to various types of inaccuracies as well as the need for special equipment and operators [10]. In lieu of these issues, several heart rate variability (HRV) indexes have been proposed as a means of defining the low-intensity threshold transition [11,12]. However, the use of time and frequency domain HRV measures has been shown to have issues with precision and reliability [13]. Recently, encouraging data have indicated that a non-linear index of fractal correlation properties of heart rate (HR) time series based on the short-term scaling exponent alpha1 of detrended fluctuation analysis (DFA a1) can indicate zone 1 transition in a population of healthy recreational athletes [14]. Whether that observation extends to other, more diverse, populations is unknown. Given the importance of proper intensity-zone assessment in cardiac rehabilitation outcome and guidance [2,3,15,16] an assessment of agreement between a DFA-a1-based HRV threshold (HRVT) with the VT1 obtained by gas exchange would be of great interest. If DFA a1 behavior is similar both in athletes and in those with cardiac disease, this could lead to more widespread interest in using this HRV index as a modality for training guidance in the low to moderate intensity areas for the purpose of intensity distribution in therapy and rehabilitation.

DFA a1 behavior during exercise has been described previously from both a theoretical and a practical standpoint [17,18]. In brief, this index represents the degree of self-similarity and fractal-like composition of a series of cardiac interbeat intervals, providing information about organismic demands and network physiology during exercise [19]. The underlying mechanism behind the change in this index with increasing exercise intensity has been attributed to alterations in the parasympathetic–sympathetic balance on the sinoatrial node as well as interactions of different organismic subsystems and (non-neural) influencing factors [12,20,21,22]. DFA a1 is a dimensionless index with a wide dynamic range centered around the VT1 [18]. At low exercise intensity, DFA a1 values remain in a well-correlated, fractal pattern that progresses to a more uncorrelated behavior past the aerobic threshold. In a previous investigation involving a diverse population of male runners, it was shown that a DFA a1 value of 0.75 reached on an incremental exercise ramp closely corresponded to the VT1 intensity measured by gas exchange [14]. A significant advantage of DFA a1 behavior as opposed to other HRV indexes used to define the aerobic threshold revolves around the underlying method of determination. Several time and frequency domain-related HRV parameters (standard deviation of NN intervals, SDNN; standard deviation 1 from Poincaré plot analysis, SD1; high frequency power of frequency domain HRV analysis, HF power) do decline with increasing exercise effort to reach a nadir near the VT1 [12]. By utilizing incremental exercise ramps, observation of when that nadir occurs can be helpful in threshold definition. However, many subjects do not display easily noted nadirs [13] and this technique requires a near-maximal-effort exercise ramp to be performed. A major benefit of DFA a1 methodology for intensity assessment is that the VT1-related threshold does not rely on nadir identification. As exercise intensity climbs, there is a near linear decline from values of 1.0 to 0.5 or below, centered around the VT1, enabling calculation of heart rate, oxygen uptake (VO_2_) or power at a predefined DFA a1 of 0.75. Further intensity increases cause continued DFA a1 decline to values well below 0.5, indicating work rates above the VT1. Therefore, the additional dynamic range provides numeric confirmation of exceeding a zone 1 target. Since the index is dimensionless, no prior normalization or ramp testing should be needed in a mixed-intensity exercise session to assess time spent in a low-intensity zone [18]. Well-established metrics of intensity such as cycling power need calibration with either gas exchange or blood lactate concentration to indicate relative load and intensity zone [10]. Although many authorities recommend intensity zones based on percentage of maximum heart rate [23], others have appropriately pointed out that these values do not necessarily match those derived from gas exchange or lactate methods [10]. Conversely, it has been shown in healthy subjects that the DFA a1 index appears to be already standardized to exercise load status, with values lower than 0.75 representing intensity above the VT1. 

Hence, the purpose of this study is to explore two major questions. First, does the DFA-a1-derived HRVT agree with the VT1 determined by gas exchange in subjects with congestive heart failure or ischemic cardiac disease? Second, does the change in HRVT correspond to the change in the VT1 after a standard cardiac rehabilitation exercise program?

## 2. Materials and Methods

### 2.1. Participants

Sixteen volunteers with either stable congestive heart failure (CHF) or previous exertional angina (CAD) were evaluated before and after an exercise rehabilitation program. All subjects underwent baseline cardiac catheterization, had an unchanged serial resting ECG and were deemed clinically stable for 3 weeks prior to each incremental exercise ramp testing session. Participants with CAD had ejection fractions above 50% and had normal cardiac contractile function. The etiology of the CHF was of either ischemic and/or dilated cardiomyopathy with ejection fractions below 40%. All participants were on beta blockade therapy except for one (Table 1). No participant had symptoms of unstable angina or evidence of significant ventricular arrythmia, atrial fibrillation, supraventricular arrhythmias or exercise-test-related difficulty. The study protocol complied with the Declaration of Helsinki and was reviewed and accepted by the ethical committee of Tours (France; no. 2005–2025). All participants were informed about the study procedure and risks and gave their written informed consent. 

### 2.2. Exercise Testing Protocol

Participants performed an incremental cycling ramp in an upright position on a cycle ergometer with careful monitoring for symptoms (ERG 900, GE Medical System, CASE Exercise Testing System Case, Milwaukee, WI, USA). Ramp protocol consisted of an initial cycling power of 20 watts followed by a 10 watt increase every minute until exhaustion. Peak effort was confirmed by failure of oxygen uptake (VO_2_) and/or HR to increase with further increases in work rate. Testing procedures were the same pre- and post-exercise intervention. 

### 2.3. Exercise Training Intervention 

The exercise training intervention consisted of cycle ergometer sessions (30 min per day, 5 times per week), at an intensity approximating the participants’ VT1 heart rate, which was measured during the first incremental ramp test. In addition, gymnastic (callisthenic) sessions of 50 min per day were performed either in a conventional fashion or immersed in a swimming pool at an intensity at or below the VT1 [24]. The participants also attended counseling sessions centered around secondary cardiovascular prevention, stress management, nutritional guidance and smoking cessation. No exercise sessions were devoted to training above VT1-related intensity.

### 2.4. Gas Exchange Testing

Gas exchange parameters were measured breath-by-breath using a Vmax Spectra system (SensorMedics Corporation, Yorba Linda, CA, USA). VO_2_, carbon dioxide output (VCO_2_) and HR were imported into Microsoft Excel 365 for detailed analysis. Cycling power was measured continuously and reported for each gas exchange measurement. Graphing of the above parameters were done to derive VT1, VO_2PEAK_ and VO_2_ vs. time. VT1 was determined by the excess CO_2_ method using the breath-by-breath data without averaging [25] with two experienced observers confirming VT1 results. If no consensus was found, an additional opinion was sought. VO_2_ at the time of VT1 was based on linear regression of the VO_2_ over time relationship excluding a VO_2_ plateau, if present. Both VO_2PEAK_ and peak cycling power (P_PEAK_) were obtained from an average of each participant’s VO_2_ or cycling power over the last 60 s of the ramp test. Power at VT1 was calculated from the 60 s average power centered at the time of VT1 [26]. Peak heart rate (HR_PEAK_) was defined as the average of the three highest heart rates measured during the final 60 s of the test.

### 2.5. RR Measurements and Calculation of DFA-a1-Derived Threshold 

An ECG system (VISTA Holter NOVACOR, Rueil, Malmaison, France) with a sampling rate of 200 Hz was used to record the participant’s ECG/RR times series. The RR data were extracted as a text file and imported into Kubios HRV Software Version 3.4.2 (Biosignal Analysis and Medical Imaging Group, Department of Physics, University of Kuopio, Kuopio, Finland). Kubios’ preprocessing settings were at the default values including the RR detrending method, which was kept at “Smoothn priors” (Lambda = 500). The DFA a1 window width was set to 4 ≤ *n* ≤ 16 beats. The RR series was then corrected by the Kubios “automatic method”, and relevant HRV parameters were exported as text files for further analysis. Percent of artifacts during pertinent measurement windows was usually less than 2% and never above 5%. DFA a1 was calculated from the RR data series using 2 min time windows with repeat computation every 5 s throughout the test. The moving time window measurement was used to better delineate rapid changes in the DFA a1 index over the course of the test. For the detection of HRVT, a DFA a1 value of 0.75 was chosen based on a previous study in recreational athletes [14]. This value is also the midpoint between a fractal, well-correlated behavior of the HR time series of 1.0 (seen with very light exercise) and an uncorrelated value of 0.5, which represents white noise, random behavior (seen with high-intensity exercise). Plotting of DFA a1 vs. time was then performed, generally showing a reverse sigmoidal curve with a stable area above 1.0 at low work rates, a rapid, near linear drop reaching below 0.5 at higher intensity, then flattening without major change. The procedure used to indicate at what level of cycling intensity (as per VO_2_ or HR) the DFA a1 would cross a value of 0.75 has been detailed previously [14]. Note should be made that the HR at DFA a1 0.75 (based on ECG data) was compared to the HR at VT1 obtained from the metabolic cart data. Power at DFA a1 = 0.75 (power at HRVT) was calculated from the 60 s average power centered at the time DFA a1 reached 0.75.

### 2.6. Statistics

Statistical analysis was performed for the key variables, VO_2PEAK_, P_PEAK_, HR_PEAK_, VO_2_, HR and power at VT1, VO_2_, HR and power at HRVT. Conventional methods were used for the calculation of means and standard deviations (SDs). Normal distribution of data was checked by Shapiro–Wilk’s testing. Agreement against the established standard VT1-related parameters was assessed using Pearson’s r correlation coefficient, standard error of estimate (SEE), coefficient of determination (R^2^) and Bland–Altman plots with limits of agreement [27]. The magnitude of Pearson’s r correlations was evaluated as follows: 0.3 ≤ r < 0.5, low; 0.6 ≤ r < 0.8, moderate and r ≥ 0.8, high [28]. Paired t-testing was used for comparison of PRE vs. POST intervention. For all tests, the statistical significance was accepted as *p* ≤ 0.05. Cohen’s d was used to denote effect sizes (small effect = 0.2, medium effect = 0.5, large effect = 0.8) [29]. Analysis was performed using Microsoft Excel 365 with Real Statistics Resource Pack software (Release 6.8) and Analyse-it Software (Version 5.66).

## 3. Results

### 3.1. Comparison of VT1 and HRVT

**Figure 1:**
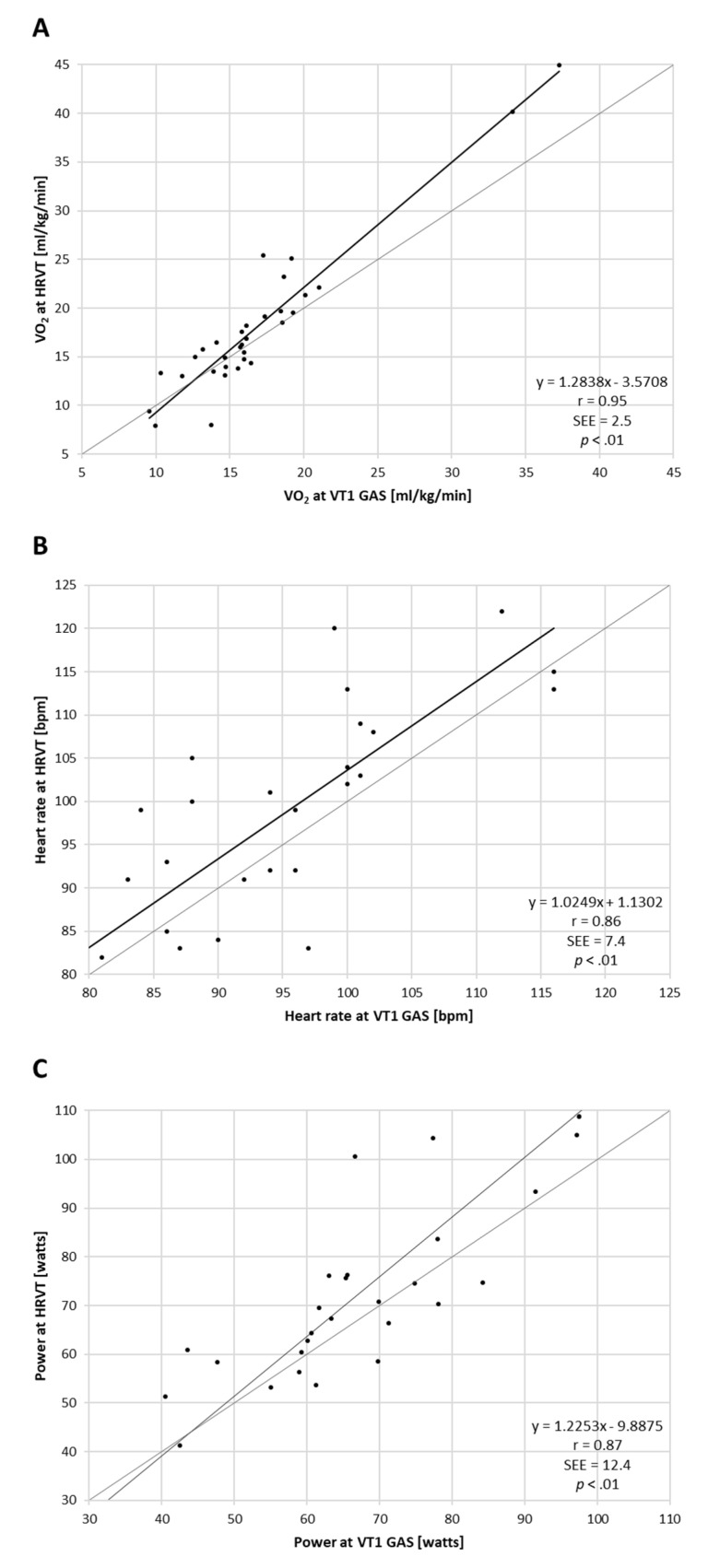
Regression plots for all participant ramp tests performed. (**A**) VT1 vs. HRVT for VO_2_; (**B**) VT1 vs. HRVT for HR; (**C**) VT1 vs. HRVT for cycling power. Bisection lines in light gray.

**Figure 2:**
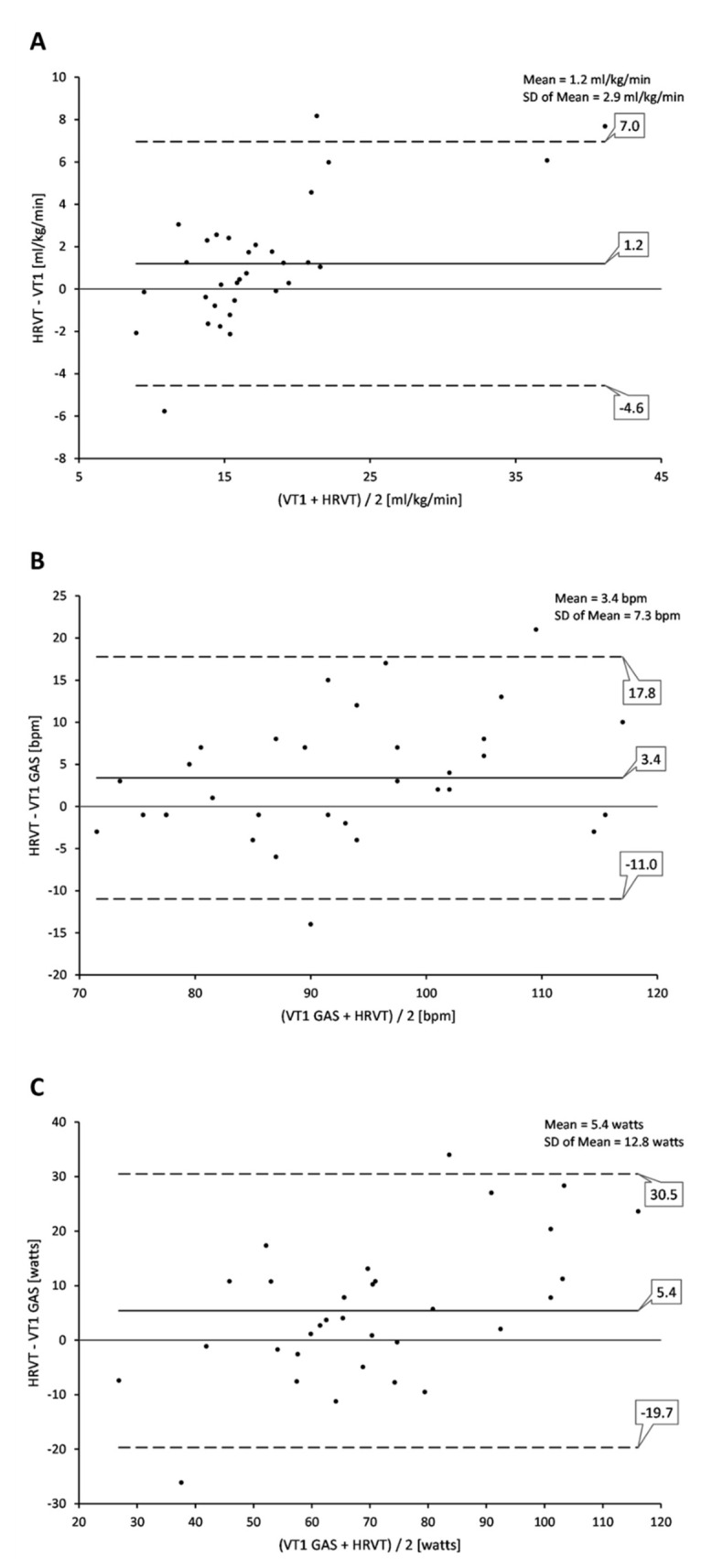
Bland–Altman plot of VT1 vs. HRVT for all participant ramp tests performed. (**A**) VT1 vs. HRVT for VO_2_; (**B**) VT1 vs. HRVT for HR; (**C**) VT1 vs. HRVT for cycling power. Center line in each plot represents the mean difference (bias) between each paired value, the top and bottom dashed lines are 1.96 standard deviations from the mean difference.

Regression analysis for comparisons of VT1 vs. HRVT (such as their respective VO_2_, HR and power values) calculated from all ramp tests performed (both PRE and POST) are shown in Figure 1. Strong correlations were seen between VT1-based determinations and those derived from HRVT. Pearson’s r was calculated at 0.95, 0.86 and 0.87 (all *p* < 0.001) for gas-based VO_2_ (mL/kg/min), HR and power comparisons, respectively. Bland–Altman evaluation for each metric comparison is shown in Figure 2. Mean differences between gas-exchange-based VT1 and HRVT were 1.20 mL/kg/min for VO_2_, 3.4 bpm for HR and 5.4 watts for power. Limits of agreement (1.96 × SD) are also listed in Figure 2. 

### 3.2. PRE vs. POST Training Comparisons

**Figure 3:**
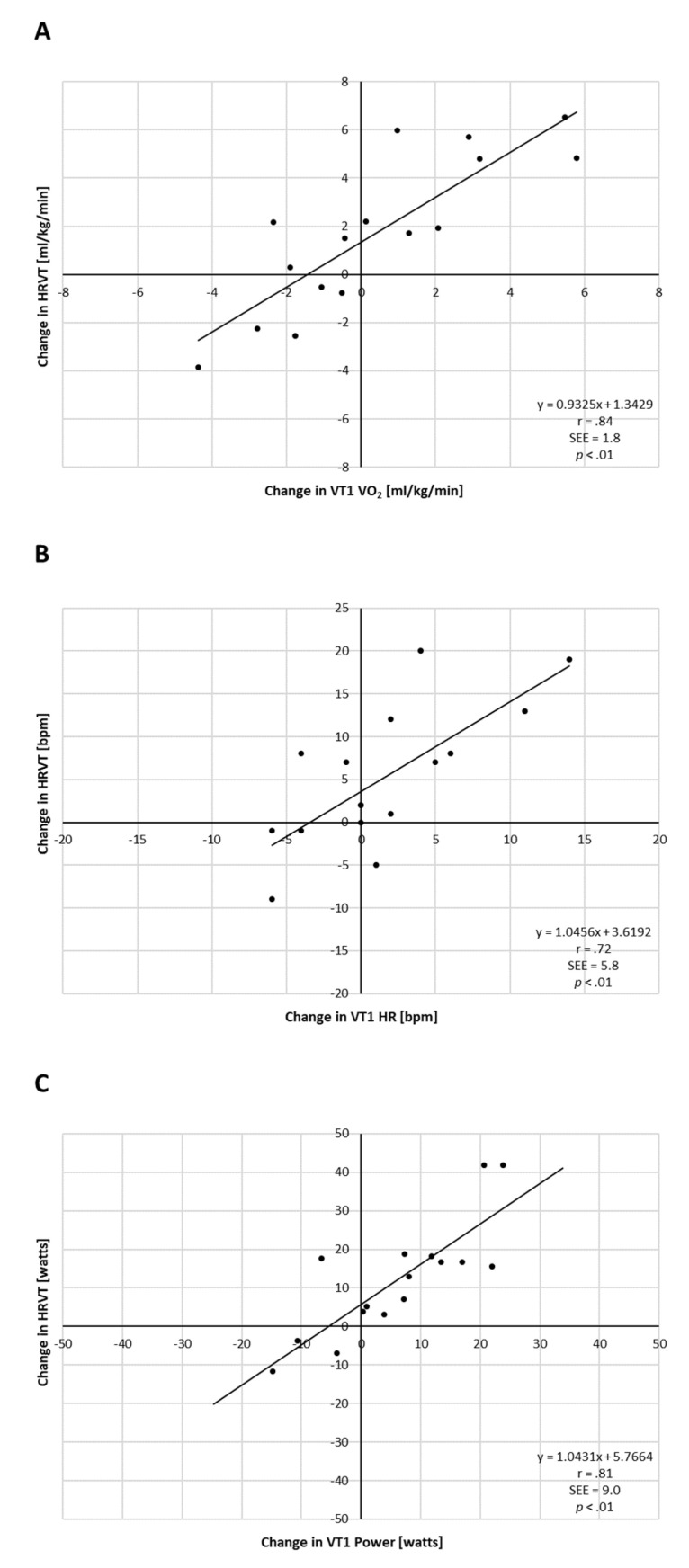
Analysis of individual improvement PRE vs. POST exercise intervention for all participants. (**A**) Change in measured VT1 vs. HRVT for VO_2_; (**B**) change in measured VT1 vs. HRVT for HR; (**C**) change in measured VT1 vs. HRVT for Power. Pearson’s r, SEE (standard error of estimate) and *p* value of regression line listed.

After exercise intervention (PRE vs. POST) HR_PEAK_ and P_PEAK_ increased markedly, which was statistically significant with high effect sizes (Table 2). VO_2PEAK_ showed no significant change. Analysis of individual changes in VT1- and HRVT-related parameters did show variation (Figure 3). Plotting the change in VT1 VO_2_ vs. the change in HRVT VO_2_ showed both the presence of heterogeneity of participant fitness response as well as a reasonable correlation of both these parameters (r = 0.84). A similar correlation was seen between both gas-exchange-based HR (r = 0.72) and power (r = 0.81) to that determined by HRVT (see Figure 3). 

## 4. Discussion

The aim of this study centered around answering two questions. First, does the DFA-a1-derived HRVT correspond with the gas-exchange-based VT1 in a population with a spectrum of cardiac disease? Second, does the change in the HRVT follow that of the VT1 after a cardiac rehabilitation exercise program? Although a recent study involving healthy men indicated that the HRVT was closely associated with the occurrence of the VT1 [14], extension of these findings should be done in other exercise types (cross country ski, rowing, swimming) and demographic groups before assuming generalization across most individuals. Patients with cardiac disease represent an important category for the investigation of intensity-zone boundary demarcation for several reasons. They are a popular target for physical exercise and training interventions to improve morbidity and mortality [16] and could benefit from non-invasive methods to measure, track and recommend exercise intensity zones. Most previous studies involving DFA a1 behavior during exercise have been done in physically fit individuals or even elite athletes [17]. The display of similar behavior in a population encompassing ischemic heart disease, heart failure and beta blockade usage would provide reassurance of its validity for more general use. The average numerical differences between the VT1 and HRVT based on VO_2_, HR or power were small. Indeed, Bland–Altman mean differences amounted to 1.2 mL/kg/min, 3.4 bpm and 5.4 watts for VO_2_, HR and cycling power. Regression analysis showed strong measures of correlation with r values 0.95, 0.86 and 0.87 for the above parameters, respectively. However, it should be noted that the limit of agreements for the listed measures were −4.6 to 7.0 mL/kg/min, −11.0 to 17.8 bpm and −19.7 to 30.5 watts, which are relatively wide. There are only few studies that have assessed heart rate variability–related thresholds in cardiac disease and none regarding DFA a1 [30,31]. These reports evaluated the association of VT1 with SD1, SDNN and HF power × HF frequency peak methods in a comparable cardiac disease population. They did show that there was a relationship of these metrics to the VT1, similar to that seen with healthy individuals. However, DFA a1 HRVT analysis does present several advantages over the other indexes. SD1 and SDNN both rely on nadir values on formal ramp testing for the determination of an HRV threshold. DFA a1 is a dimensionless index with its center of dynamic range near the VT1/aerobic threshold. This provides an opportunity to identify the HRVT more easily as a nadir does not need to be demonstrated. Since asymptotic curve behavior is not needed, there is potential to be able to show low-intensity-zone adherence in a free-form exercise training session [18]. Given its dimensionless nature, calibration with gas exchange should not be needed. Therefore, the DFA a1 threshold of 0.75 can potentially be observed over an exercise session, enabling subjects to track and limit their intensity accordingly in a real-time monitoring assessment [32].

The second major finding of this report is that the post-exercise intervention change in HRVT did relate to the change in VT1 but not to VO_2PEAK_. Although there was little net improvement in post-exercise VO_2PEAK_ or VT1 for the entire group, there were individual changes in the various fitness parameters with the modest exercise training intervention employed. The lack of VO_2PEAK_ increase post training has been reported previously [33], although there is evidence for improvement in other studies employing high-intensity sessions [3]. In our study group, the lack of net VO_2PEAK_ improvement should not be unexpected given extensive population studies examining the inconsistent ability of exercise to improve this parameter [34]. Additionally, the exercise protocol used here did not have a high-intensity component, which has been shown to promote VO_2PEAK_ enhancement sessions [3]. The change in HRVT measurement for VO_2_, HR or power occurred in parallel to that of the change in gas-exchange-based data, demonstrating the usefulness of fractal HR dynamics to longitudinally follow the aerobic threshold. This would be valuable from multiple standpoints including adjustment of training zones, estimating intervention success and pointing out potential non-responders to a given exercise protocol. Lastly, there is little information on DFA-a1-based HRVT retest reliability. The strong correlation of HRVT with VT1 both pre- and post-exercise intervention does support the retest consistency of an individual’s DFA a1 response to exercise intensity.

The mechanism behind many HRV-related aerobic threshold determinations, including DFA a1, appears to be related to the reciprocal antagonistic behavior and the interaction of the sympathetic and parasympathetic branch of the autonomic nervous system, with an increase of the former and withdrawal of the latter as exercise intensity rises [12,20,22]. Since all participants but one in this study were using beta adrenergic blockade, the fact that their HRVT behavior was comparable to that of a population of non-medicated individuals, argues for the importance of parasympathetic withdrawal as the major influencing factor.

## 5. Limitations and Future Directions

Participants in this study comprised a mixed group with diagnoses of both ischemic cardiac disease and heart failure. Subgroup investigations of heart-failure- vs. ischemic-disease-related HRVT accuracy were not done due to the low sample size. An in-depth look at the HRVT in homogenous groups with heart failure or ischemic vascular disease or even those with cardiac valvular disease would be ideal. Additionally, no female patients were evaluated, which is an area that needs exploration since few studies examining DFA a1 behavior in women exist. While it appears that beta adrenergic blockade therapy does not affect HRVT determination, we did not have a comparison group of patients without this medication. The basis for assuming the lack of effect of beta blockade on the HRVT centers around the similarity of the results presented here and that seen in healthy subjects [14]. In support of this finding, an investigation of beta blockade effects on detrended scaling properties of HRV at rest showed no major impact at short time scales such as DFA a1 [35]. However, the interaction of beta blockade usage in individuals with coexistent cardiac disease could yield different results. Correlation properties of HRV in patients with ischemic cardiac disease or heart failure, measured at rest, have been linked to mortality [36]. In addition, the use of beta blockade therapy was shown to raise (improve) resting DFA a1 values toward more fractal-like behavior [37]. The issue of recording device sample rate should also be raised. The ECG recording unit employed here was a medical-grade Holter device, with a sample rate of 200 Hz, lower than many recommendations [38]. A previous study [39] did find a small but significant 3% difference between DFA a1 values at rest comparing 128 vs. 500 Hz sampling devices. Thus, although potential bias should be minimal, further research could explore sample-rate-related effects on the HRVT. Artifact-correction-related issues with DFA a1 have been studied [40], and recently our group reported minimal effects on the HRVT with missed beat artifact below 6% [41]. Fortunately, in the current study, Kubios software artifact estimation was below 5% and largely below 2% during the HRVT calculation. Although the limits of agreement between the HRVT and gas exchange metrics were wider than those of a previous study of DFA a1 [14], they were similar to other comparisons to the VT1 such as with lactate markers [42].

With a look to the future of DFA-a1-guided intensity training, the most practical issue is whether ramp-derived HRVT corresponds with that of DFA a1 behavior during constant power intervals. If it can be shown that the DFA a1 cutoff of 0.75 is a robust marker for zone 1 to 2 transition throughout the course of a free-form exercise session, this would open the way for both real-time and retrospective assessment of exercise session intensity. Although both HR and power metrics are readily available, without prior standardization to either gas exchange or lactate testing (for instance, HR or power at VT1 or LT1), they are poor guides to relative intensity, especially the aerobic threshold [10]. DFA a1 is a dimensionless index, already “calibrated” to external load due to the expression of fractal patterns of HR time series as a proxy of organismic demands and internal load of the entire system [18]. Although more investigation needs to be done regarding usage over longer time frames, values above the 0.75 level should correspond to intensities below the aerobic threshold and suitable for zone 1 training. With chest belt recording devices, smartphone or smartwatch form factors and appropriate software already available, real-time tracking of DFA a1 could aid with both aerobic threshold estimation as well as enforcement of a low-intensity limit in cardiac rehabilitation and athletic training [32].

## 6. Conclusions

A heart rate variability threshold based on fractal correlation properties, DFA a1, was closely related to the first ventilatory threshold in a group of individuals with either congestive heart failure or ischemic vascular disease. Although there was a small difference in VO_2_, heart rate and cycling power between modalities, they were minimal from a clinical and practical standpoint. In addition, exercise-training-associated change in the first ventilatory threshold strongly agreed with the change seen in the heart rate variability threshold. Therefore, even though the improvement of the first ventilatory threshold was not universal, a non-invasive measure was able to predict that change. It also appears that the usage of beta-adrenergic blocker therapy did not alter the relationship of the heart rate variability threshold to the first ventilatory threshold and its application in exercise therapy. This is important for individuals on these medications and also indicates that parasympathetic-withdrawal-associated loss of correlation properties is one of the predominate mechanisms for DFA a1 decline during exercise.

## Figures and Tables

**Table 1:** Demographic data of all participants (*n* = 16).

Age (Years)	Ht (cm)	BW (kg)	HR_PEAK_ (bpm)	VO_2PEAK_ (mL/kg/min)	Etiology
52	173	71	117	17.36	CHF
59	168	73	109	23.05	CAD
55	176	89	141	31.42	CAD
62	172	85	129	28.96	CAD
56	167	72	131	42.50	CAD
55	176	94	112	24.84	CAD
54	178	94	95	25.89	CAD
59	163	58	110	24.93	CAD
41	176	58	128	51.23	CAD
53	171	96	131	27.21	CHF
44	173	67	133	33.05	CAD
70	174	81	108	21.52	CHF
64	170	74	114	35.67	CHF
40	182	89	162	27.88	CHF *
62	175	97	132	28.53	CAD
58	175	67	131	22.28	CHF
55 (±8)	173 (±5)	79.0 (±13)	124 (±16)	29.15 (±8.42)	-

Age: current age, Ht: height, BW: body weight, HR_PEAK_: peak heart rate on baseline ramp test, VO_2PEAK_: peak oxygen uptake reached on baseline ramp test, Etiology: cause of cardiac disease, either congestive heart failure (CHF) or ischemic coronary artery disease (CAD) (* denotes no beta blocker therapy usage); mean (± standard deviation, SD) in last row.

**Table 2:** PRE and POST exercise intervention test data of all participants (*n* = 16).

	PRE	POST	Paired t Testing and Effect Size
BW (kg)	79.0 (±13.0)	78.1 (±12.5)	*p* = 0.03, d = 0.61
HR_PEAK_ (bpm)	124 (±16)	135 (±18)	*p* < 0.01, d = 1.66
VO_2PEAK_ (mL/kg/min)	29.15 (±8.42)	30.73 (±10.01)	*p* = 0.07, d = 0.49
VT1 VO_2_ (mL/kg/min)	16.61 (±5.54)	17.02 (±6.06)	*p* = 0.58, d = 0.14
HRVT VO_2_ (mL/kg/min)	17.15 (±7.61)	18.88 (±8.02)	*p* = 0.05, d = 0.54
VT1 HR (bpm)	90.5 (±11.7)	92.0 (±12.3)	*p* = 0.30, d = 0.27
HRVT HR (bpm)	92.1 (±13.6)	97.3 (±14.7)	*p* = 0.02, d = 0.64
P_PEAK_ (watts)	119.5 (±29.0)	135.8 (±34.0)	*p* < 0.01, d = 1.92
VT1 P (watts)	64.7 (±18.2)	70.9 (±17.5)	*p* = 0.05, d = 0.54
HRVT P (watts)	67.1 (±25.1)	79.4 (±24.2)	*p* < 0.01, d = 0.82

BW: body weight, HR_PEAK_: peak heart rate, VO_2PEAK_: peak oxygen uptake, VT1 VO_2_: oxygen uptake at first ventilatory threshold; HRVT VO_2_: oxygen uptake at heart rate variability threshold, VT1 HR: heart rate at first ventilatory threshold, HRVT HR: heart rate at the heart rate variability threshold, P_PEAK_: peak cycling power, VT1 P: average power at first ventilatory threshold, HRVT P: average power at heart rate variability threshold; mean (± standard deviation, SD) listed; *p*-values: result of two-tailed paired t-testing; Cohen’s d: effect size for each significant comparison.

## Data Availability

The raw data supporting the conclusions of this article will be made available by the authors, without undue reservation. The data are not publicly available due to logistical reasons.

## References

[1] Bourgois J.G., Bourgois G., Boone J. (2019). Perspectives and Determinants for Training-Intensity Distribution in Elite Endurance Athletes. Int. J. Sports Physiol. Perform..

[2] Mezzani A., Hamm L.F., Jones A.M., McBride P.E., Moholdt T., Stone J.A., Williams M.A. (2013). Aerobic exercise intensity assessment and prescription in cardiac rehabilitation: A joint position statement of the European Association for Cardiovascular Prevention and Rehabilitation, the American Association of Cardiovascular and Pulmonary Rehabilitation and the Canadian Association of Cardiac Rehabilitation. Eur. J. Prev. Cardiol..

[3] Hannan A.L., Hing W., Simas V., Climstein M., Coombes J.S., Jayasinghe R., Furness J. (2018). High-intensity interval training versus moderate-intensity continuous training within cardiac rehabilitation: A systematic review and meta-analysis. Open Access. J. Sports Med..

[4] Marcin T., Eser P., Prescott E., Prins L.F., Kolkman E., Bruins W., van der Velde A.E., Gil C.P., Iliou M.-C., Ardissino D. (2020). Training intensity and improvements in exercise capacity in elderly patients undergoing European cardiac rehabilitation—The EU-CaRE multicenter cohort study. PLoS ONE.

[5] Seiler K.S., Kjerland G.Ø. (2006). Quantifying training intensity distribution in elite endurance athletes: Is there evidence for an “optimal” distribution?. Scand. J. Med. Sci. Sports.

[6] Esteve-Lanao J., Foster C., Seiler S., Lucia A. (2007). Impact of training intensity distribution on performance in endurance athletes. J. Strength Cond. Res..

[7] Meyer K., Hajric R., Westbrook S., Samek L., Lehmann M., Schwaibold M., Roskamm H. (1996). Ventilatory and lactate threshold determinations in healthy normals and cardiac patients: Methodological problems. Eur. J. Appl. Physiol. Occupat. Physiol..

[8] Faude O., Kindermann W., Meyer T. (2009). Lactate threshold concepts. Sports Med..

[9] Jamnick N.A., Botella J., Pyne D.B., Bishop D.J. (2018). Manipulating graded exercise test variables affects the validity of the lactate threshold and VO2peak. PLoS ONE.

[10] Jamnick N.A., Pettitt R.W., Granata C., Pyne D.B., Bishop D.J. (2020). An Examination and Critique of Current Methods to Determine Exercise Intensity. Sports Med..

[11] Karapetian G.K., Engels H.J., Gretebeck R.J. (2008). Use of heart rate variability to estimate LT and VT. Int. J. Sports Med..

[12] Michael S., Graham K.S., Davis Oam G.M. (2017). Cardiac Autonomic Responses during Exercise and Post-exercise Recovery Using Heart Rate Variability and Systolic Time Intervals-A Review. Front. Physiol..

[13] Blasco-Lafarga C., Camarena B., Mateo-March M. (2017). Cardiovascular and Autonomic Responses to a Maximal Exercise Test in Elite Youngsters. Int. J. Sports Med..

[14] Rogers B., Giles D., Draper N., Hoos O., Gronwald T. (2021). A New Detection Method Defining the Aerobic Threshold for Endurance Exercise and Training Prescription Based on Fractal Correlation Properties of Heart Rate Variability. Front. Physiol..

[15] Carvalho V.O., Mezzani A. (2011). Aerobic exercise training intensity in patients with chronic heart failure: Principles of assessment and prescription. Eur. J. Cardiovasc. Prev. Rehabil..

[16] Franklin B.A., Lavie C.J., Squires R.W., Milani R.V. (2013). Exercise-based cardiac rehabilitation and improvements in cardiorespiratory fitness: Implications regarding patient benefit. Mayo Clin. Proc..

[17] Gronwald T., Hoos O. (2020). Correlation properties of heart rate variability during endurance exercise: A systematic review. Ann. Noninvasive Electrocardiol..

[18] Gronwald T., Rogers B., Hoos O. (2020). Fractal correlation properties of heart rate variability: A new biomarker for intensity distribution in endurance exercise and training prescription?. Front. Physiol..

[19] Balagué N., Hristovski R., Almarcha M., Garcia-Retortillo S., Ivanov P.C. (2020). Network Physiology of Exercise: Vision and Perspectives. Front. Physiol..

[20] Tulppo M.P., Kiviniemi A.M., Hautala A.J., Kallio M., Seppänen T., Mäkikallio T.H., Huikuri H.V. (2005). Physiological background of the loss of fractal heart rate dynamics. Circulation.

[21] Voss A., Schulz S., Schroeder R., Baumert M., Caminal P. (2009). Methods derived from nonlinear dynamics for analysing heart rate variability. Philos. Trans. R. Soc. A Math. Phys. Eng. Sci..

[22] White D.W., Raven P.B. (2014). Autonomic neural control of heart rate during dynamic exercise: Revisited. J. Physiol..

[23] American College of Sports Medicine (2021). Acsm’s Guidelines for Exercise Testing and Prescription.

[24] Teffaha D., Mourot L., Vernochet P., Ounissi F., Regnard J., Monpère C., Dugué B. (2011). Relevance of water gymnastics in rehabilitation programs in patients with chronic heart failure or coronary artery disease with normal left ventricular function. J. Card. Fail..

[25] Gaskill S.E., Ruby B.C., Walker A.J., Sanchez O.A., Serfass R.C., Leon A.S. (2001). Validity and reliability of combining three methods to determine ventilatory threshold. Med. Sci. Sports. Exerc..

[26] Bentley D.J., McNaughton L.R. (2003). Comparison of W(peak), VO2(peak) and the ventilation threshold from two different incremental exercise tests: Relationship to endurance performance. J. Sci. Med. Sport.

[27] Bland J.M., Altman D.G. (1999). Measuring agreement in method comparison studies. Stat. Methods Med. Res..

[28] Chan Y.H. (2003). Biostatistics 104: Correlational analysis. Singap. Med. J..

[29] Cohen J. (1988). Statistical Power Analysis for the Behavioral Sciences.

[30] Mourot L., Tordi N., Bouhaddi M., Teffaha D., Monpere C., Regnard J. (2012). Heart rate variability to assess ventilatory thresholds: Reliable in cardiac disease?. Eur. J. Prev. Cardiol..

[31] Leprêtre P.M., Bulvestre M., Ghannem M., Ahmaidi S., Weissland T., Lopes P. (2013). Determination of Ventilatory Threshold using Heart Rate Variability in Patients with Heart Failure. Surgery.

[32] Gronwald T., Berk S., Altini M., Mourot L., Hoos O., Rogers B. (2021). Real-Time Estimation of Aerobic Threshold and Exercise Intensity Distribution Using Fractal Correlation Properties of Heart Rate Variability: A Single-Case Field Application in a Former Olympic Triathlete. Front. Sports Act. Living.

[33] Nichols S., Taylor C., Goodman T., Page R., Kallvikbacka-Bennett A., Nation F., Ingle L. (2020). Routine exercise-based cardiac rehabilitation does not increase aerobic fitness: A CARE CR study. Int. J. Cardiol..

[34] Bouchard C., An P., Rice T., Skinner J.S., Wilmore J.H., Gagnon J., Rao D.C. (1999). Familial aggregation of VO(2max) response to exercise training: Results from the HERITAGE Family Study. J. Appl. Physiol..

[35] Castiglioni P., Parati G., Di Rienzo M., Carabalona R., Cividjian A., Quintin L. (2011). Scale exponents of blood pressure and heart rate during autonomic blockade as assessed by detrended fluctuation analysis. J. Physiol..

[36] Huikuri H.V., Stein P.K. (2013). Heart rate variability in risk stratification of cardiac patients. Prog. Cardiovasc. Dis..

[37] Ridha M., Mäkikallio T.H., Lopera G., Pastor J., De Marchena E., Chakko S., Myerburg R.J. (2002). Effects of carvedilol on heart rate dynamics in patients with congestive heart failure. Ann. Noninvasive Electrocardiol..

[38] Merri M., Farden D.C., Mottley J.G., Titlebaum E.L. (1990). Sampling frequency of the electrocardiogram for spectral analysis of the heart rate variability. IEEE Trans. Biomed. Eng..

[39] Tapanainen J.M., Seppänen T., Laukkanen R., Loimaala A., Huikuri H.V. (1999). Significance of the Accuracy of RR Interval Detection for the Analysis of New Dynamic Measures of Heart Rate Variability. Ann. Noninvasive Electrocardiol..

[40] Rincon Soler A.I., Silva L.E.V., Fazan R., Murta L.O. (2018). The impact of artefact correction methods of RR series on heart rate variability parameters. J. Appl. Physiol..

[41] Rogers B., Giles D., Draper N., Mourot L., Gronwald T. (2021). Influence of Artefact Correction and Recording Device Type on the Practical Application of a Non-Linear Heart Rate Variability Biomarker for Aerobic Threshold Determination. Sensors.

[42] Pallarés J.G., Morán-Navarro R., Ortega J.F., Fernández-Elías E., Mora-Rodriguez R. (2016). Validity and reliability of ventilatory and blood lactate thresholds in well-trained cyclists. PLoS ONE.

